# *Notice to Readers:* Change in Publication Date of QuickStats

**DOI:** 10.15585/mmwr.mm7313a3

**Published:** 2024-04-04

Effective this issue, *MMWR* will publish QuickStats on a bimonthly basis, in the first and third issues of each month. QuickStats will continue to present concise data from CDC’s National Center for Health Statistics on a wide range of timely and important health topics. To supplement QuickStats, a detailed table will be posted in CDC Stacks and linked to each QuickStats.

